# The potential of federated learning for self-configuring medical object detection in heterogeneous data distributions

**DOI:** 10.1038/s41598-024-74577-0

**Published:** 2024-10-11

**Authors:** Gabriel Rashidi, Dimitrios Bounias, Markus Bujotzek, Andrés Martínez Mora, Peter Neher, Klaus H. Maier-Hein

**Affiliations:** 1 German Cancer Research Center (DKFZ) Heidelberg, Division of Medical Image Computing, Heidelberg, 69120 Germany; 2 Faculty of Mathematics and Computer Science, Heidelberg University, Heidelberg, 69120 Germany; 3 Medical Faculty Heidelberg, Heidelberg University, Heidelberg, 69120 Germany; 4 German Cancer Consortium (DKTK), Partner Site Heidelberg, Im Neuenheimer Feld 280, Heidelberg, 69120 Germany; 5 Pattern Analysis and Learning Group, Heidelberg University Hospital, Heidelberg, 69120 Germany; 6 National Center for Tumor Diseases (NCT), Heidelberg University Hospital (UKHD) and German Cancer Research Center (DKFZ), Im Neuenheimer Feld 460, Heidelberg, 69120 Germany

**Keywords:** Computer science, Information technology, Medical imaging, Image processing, Machine learning

## Abstract

Medical Object Detection (MOD) is a clinically relevant image processing method that locates structures of interest in radiological image data at object-level using bounding boxes. High-performing MOD models necessitate large datasets accurately reflecting the feature distribution of the corresponding problem domain. However, strict privacy regulations protecting patient data often hinder data consolidation, negatively affecting the performance and generalization of MOD models. Federated Learning (FL) offers a solution by enabling model training while the data remain at its original source institution. While existing FL solutions for medical image classification and segmentation demonstrate promising performance, FL for MOD remains largely unexplored. Motivated by this lack of technical solutions, we present an open-source, self-configuring and task-agnostic federated MOD framework. It integrates the FL framework Flower with nnDetection, a state-of-the-art MOD framework and provides several FL aggregation strategies. Furthermore, we evaluate model performance by creating simulated Independent Identically Distributed (IID) and non-IID scenarios, utilizing the publicly available datasets. Additionally, a detailed analysis of the distributions and characteristics of these datasets offers insights into how they can impact performance. Our framework’s implementation demonstrates the feasibility of federated self-configuring MOD in non-IID scenarios and facilitates the development of MOD models trained on large distributed databases.

## Introduction

Medical object detection (MOD) is a crucial component of medical image analysis, alongside medical image classification and segmentation. Unlike segmentation, which operates at pixel level, MOD works at object level. It serves as a rapid diagnostic tool for localizing and categorizing structures like organs and lesions, being less susceptible to interrater variability^1^ compared to segmentation. As such, MOD can be an essential addition to improve diagnostic efficiency and accuracy.

High-performing MOD models require large datasets that cover a large feature space. However, strict data privacy regulations such as the Health Insurance Portability and Accountability Act (HIPAA) in the US or the General Data Protection Regulation (GDPR) in the EU often hinder the consolidation of such datasets. This causes the scarcity of training data, which negatively affects model performance and generalization.

Federated Learning (FL)^2^ offers a solution to the described problem by implementing a decentralized alternative to the conventional centralized approach. With FL, the data remain at their original source, enabling collaborative training with large distributed datasets, even under strict privacy regulations.

Most research contributions regarding image processing in FL focus on the general field of computer vision (CV) outside the medical field. For example, FedCV^3^ implements a flexible and efficient FL framework for CV tasks such as classification, segmentation, and object detection. It supports various FL strategies, including FedAvg^2^ and FedProx^4^, while investigating the effects of data domain shifts on model performance. However, CV models cannot be directly applied to MOD as medical images differ from general images. Some notable differences include unique characteristics such as varying resolutions, noise levels, and artifacts depending on the imaging modality. Additionally, severe class imbalances arise from the infrequency of small objects or specific anomalies, introducing further significant distinctions.

Existing examples of applying FL in the medical field include Kades et al.^5^, who created a federated self-configuring segmentation framework based on nnU-Net^6^ or Adnan et al.^7^ addressing privacy concerns and data domain shifts in federated medical image classification. Studies such as those by Parekh et al. and Narayanan et. al.^8,9^ take a first step in investigating FL for MOD. However, they lack the application of object detection-specific model architectures such as Retina U-Net^10^. Furthermore, there is a need for a comprehensive exploration of the impact of data domain shifts on MOD, caused by FL’s multicentric model training.

**Fig. 1:**
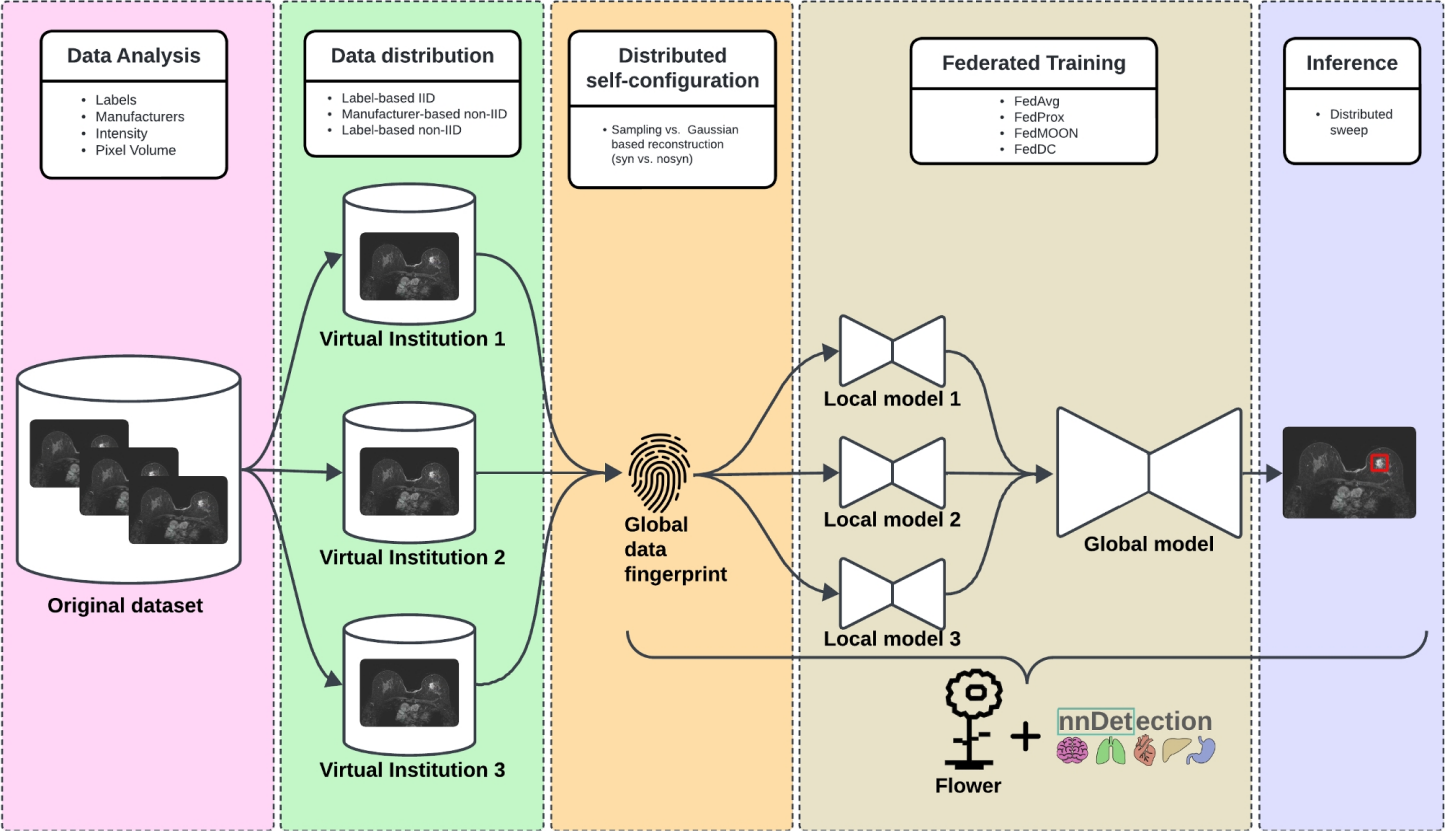
Overview of the components comprising the proposed federated self-configuring MOD framework. The data analysis component (red) enables data distribution (green) based on features such as device manufacturers and labels. As part of the self-configuration process, each virtual institution generates a local data fingerprint, which is then aggregated into a common global data fingerprint (orange). The global data fingerprint can be generated using a non-synthetic (nosyn) or synthetic (syn) method. When using the nosyn method voxel values are sampled from each virtual institution and sent to the server to compute the intensity value distribution. The syn methods avoids sampling voxel values from virtual institutions to improve patient data privacy. Details can be found in Section Global Data Fingerprint. Utilizing the global data fingerprint, each virtual institution automatically selects relevant parameters and initializes a local model for federated training (brown). After training, a distributed automatic object detection hyperparameter optimization is conducted at each local institution based on the global model to improve the model’s performance for inference (blue).

In this work, we introduce the first self-configuring FL framework for object detection in medical imaging as described in Fig. 1. This comprehensive, end-to-end solution is designed to train robust models, effectively overcoming challenges associated with data scarcity. Using Flower^11^, a lightweight FL framework, we extend nnDetection^12^, a state-of-the-art self-configuring MOD framework, into a federated self-configuring MOD framework.

One of the major concerns in FL is the feature distribution across different datasets, which impacts the performance of FL models. Heterogeneously distributed data present a significant challenge in FL as it can negatively impact model performance. To address this, we evaluate the first application of MOD models in FL, employing various FL aggregation strategies such as FedProx, FedMOON^13^, and FedDC^14^, alongside the conventional FedAvg approach. The objective is to evaluate the performance of these strategies in varying data distribution scenarios and alleviate potential adverse effects arising from data domain shifts that are common in real-world scenarios.

The detection performance evaluation of our federated MOD framework is conducted using the LUng Nodule Analysis 2016 datasets (Luna16)^15^ and the Duke Breast Cancer MRI dataset (Duke)^16–18^. These two datasets contain images from different modalities, providing a basis for demonstrating the capabilities of the proposed self-configuring FL framework. Based on extensive data analysis, the original datasets are distributed among virtual institutions in an Independent and Identically Distributed (IID) manner to assess peak performance under ideal conditions. Additionally, non-IID distributions are employed to simulate real-world conditions with heterogeneous data distributions.

The results of these experiments confirm the feasibility of utilizing FL for self-configuring MOD. We demonstrate the possibility of developing advanced detection tools while preserving data privacy and identify challenges related to the distributed self-configuration process and dataset distribution, providing valuable insights into potential areas for future research.

## Experiments

### Centralized vs. label-based IID FedAvg

The objective of this experiment is to assess FL’s optimal performance under ideal conditions, and to establish a baseline for comparison with FedAvg’s performance in non-IID scenarios. It compares the centralized nnDetection framework trained on the original consolidated dataset to our FL framework trained on a label-based IID data distribution of the original dataset using FedAvg.

In the FL experiments, we establish virtual institutions and allocate data to them, while ensuring similar label distributions among them. To explore the amount of patient data required for the distributed self-configuration process resulting in well-performing models, we evaluate two distinct data fingerprinting methods for FL training. The *syn* experiment employs a less precise global data fingerprint but avoids transferring intensity values from institutions to the server. Conversely, the *nosyn* approach relies on sampled intensity values from the individual institutions (detailed in Section Global Data Fingerprint).

### Non-IID FedAvg vs. alternative FL strategies

Considering that IID data distributions are uncommon in real-world settings^19^, we evaluate the performance of FL models under simulated real-world conditions using experiments with heterogeneous (non-IID) data distributions. These are based on variables like labels and manufacturers, introducing domain shifts that may impact model convergence and performance^20–22^.

Alternative federated learning strategies - FedProx, FedMOON, and FedDC - are compared to FedAvg, as they are expected to alleviate the adverse effects of non-IID data distributions. As FL strategies such as FedProx and FedMOON incorporate the hyperparameter $\mu$, an extensive hyperparameter optimization is conducted with different settings for $\mu$. In total, eight and ten different settings are tested for FedProx and FedMOON respectively. Table 1 summarizes the different experiments conducted for the two datasets and the different data distributions, including only the best performing settings for FedProx and FedMOON.

#### Manufacturer-based non IID

The device manufacturer can be a great source of image variability due to the application of different acquisition protocols^23^. Models trained exclusively on images from one manufacturer struggle to generalize effectively when applied to a dataset originating from a different manufacturer^24^. Considering that various institutions may employ devices from distinct manufacturers, we use the device manufacturer information to create a manufacturer-based non-IID data distribution. In this scenario, each virtual institution includes training and test images sourced from a subset of all available manufacturers. Additionally, as a result of the uneven number of images associated with specific manufacturers, this data distribution also creates a quantity skew among the different datasets.

#### Label-based non-IID

Another non-IID distribution tested resembles the label imbalance frequently observed in real-world scenarios, where the distribution of labels and classes varies across different datasets. We denote this as the “label-based non-IID” distribution, mimicking different prevalence levels of a certain diseases across different sites. When local models are trained on datasets dominated by a particular label, they tend to exhibit bias toward the majority label, often resulting in challenges in achieving satisfactory performance on samples with minority class labels.

As the Duke dataset comprises only malignant cases, the label-based non-IID is not possible and it is only done for the Luna16 dataset. In contrast to the manufacturer-based non-IID distribution, where each virtual institution receives an individual test set, a fourth institution holds all the test data and serves as test instance. This “leave-one-out” (LOO) experiment represents the case, where the global model is evaluated on a dataset containing images with a domain shift that has not been present in the training datasets. This scenario particularly emphasizes the model’s domain generalization capabilities, assessing its effectiveness in handling data characterized by unfamiliar data distributions.

Similar to Li et al.^19^ we use the Dirichlet distribution $Dir(\alpha )$ to allocate labels unevenly across different datasets. In this context, the k-dimensional vector $\alpha$ serves as the concentration parameter, delineating the components of the probability vector. For each label, a sample $X = (X_1, \ldots , X_i, \ldots , X_k) \sim \textrm{Dir}(\alpha )$ is generated, where $X_i$ represents the fraction of a specific label assigned to a training set. Malignant and non-malignant cases are allocated with $\alpha = [2000, 800, 200]$ and $\alpha = [200, 800, 2000]$ for institutions I1, I2, and I3, respectively.

**Table 1:** Overview of the experiments for FedAvg and alternative FL strategies with various hyperparameter configurations.

Strategy	Experiment	Description	Luna16	Duke
FedAvg	syn	Intensity properties based on sampled voxels	*IID*	*IID*
	nosyn	Estimated intensity properties (see Section Global Data Fingerprint)	*IID*, *non-IID*	*IID*, *non-IID*
FedProx	mu1	$\mu =1.0$	*non-IID*	*non-IID*
	mu0-01	$\mu =0.01$	*non-IID*	*non-IID*
	mu-every2nd	$${\left\{ \begin{array}{ll} \mu -= 0.1, & \ell _{r}< \ell _{r-1} \wedge \ell _{r-1} < \ell _{r-2} \\ \mu +=0.1, & \text {else} \end{array}\right. }$$	*non-IID*	*non-IID*
	mu-every-exp	$${\left\{ \begin{array}{ll} \mu /= 2, & \ell _{r} < \ell _{r-1} \\ \mu *=2, & \text {else} \end{array}\right. }$$	*non-IID*	*non-IID*
	mu-every2nd-exp	$${\left\{ \begin{array}{ll} \mu /= 2, & \ell _{r}< \ell _{r-1} \wedge \ell _{r-1} < \ell _{r-2} \\ \mu *=2, & \text {else} \end{array}\right. }$$	*non-IID*	*non-IID*
FedMOON	mu10	$\mu =10.0, \tau =0.5$	*non-IID*	*non-IID*
	mu1	$\mu =1.0, \tau =0.5$	*non-IID*	*non-IID*
	mu-every-exp	$${\left\{ \begin{array}{ll} \mu /= 2, & \ell _{r} < \ell _{r-1} \\ \mu *=2, & \text {else} \end{array}\right. }$$	*non-IID*	*non-IID*
	mu-every2nd	$${\left\{ \begin{array}{ll} \mu -= 0.1, \tau =0.5, & \ell _{r}< \ell _{r-1} \wedge \ell _{r-1} < \ell _{r-2} \\ \mu +=0.1, \tau =0.5, & \text {else} \end{array}\right. }$$	*non-IID*	*non-IID*
FedDC	b5	$${\left\{ \begin{array}{ll} \text {Daisy Chaining}, & r \% 5 = 0 \\ \text {FedAvg}, & \text {else} \end{array}\right. }$$	*non-IID*	*non-IID*

The table indicates what kind of experiment was conducted (IID and non-IID) for the corresponding FL strategy. IID experiments were conducted exclusively for FedAvg, as FL strategies such as FedProx, FedMOON, and FedDC were specifically developed to improve model performance in non-IID scenarios compared to FedAvg. For the Duke dataset, a label-based non-IID data distribution is not feasible due to the presence of only one label in the dataset.

## Results

Distributing a dataset in both IID and non-IID fashions requires a thorough investigation into its inherent characteristics. This analysis also offers insights into the degree of heterogeneity among the resulting subsets. Consequently, for each dataset, we first conduct a comprehensive analysis of its characteristics, followed by an evaluation of the model performance. Details regarding the datasets can be found in Section Datasets in Methods.

### LUng Nodule Analysis 2016 dataset

Federated training requires the distribution of the original dataset into smaller disjoint subsets for IID and non-IID scenarios. The size and features of the Luna16 dataset facilitate label-based IID, manufacturer-based non-IID, and leave-one-out (LOO) label-based non-IID distributions for three distinct institutions. Although the individual local training and test datasets vary across different data distributions, the original training and test datasets remain consistent for each data distribution.

#### Data distribution comparison

**Fig. 2:**
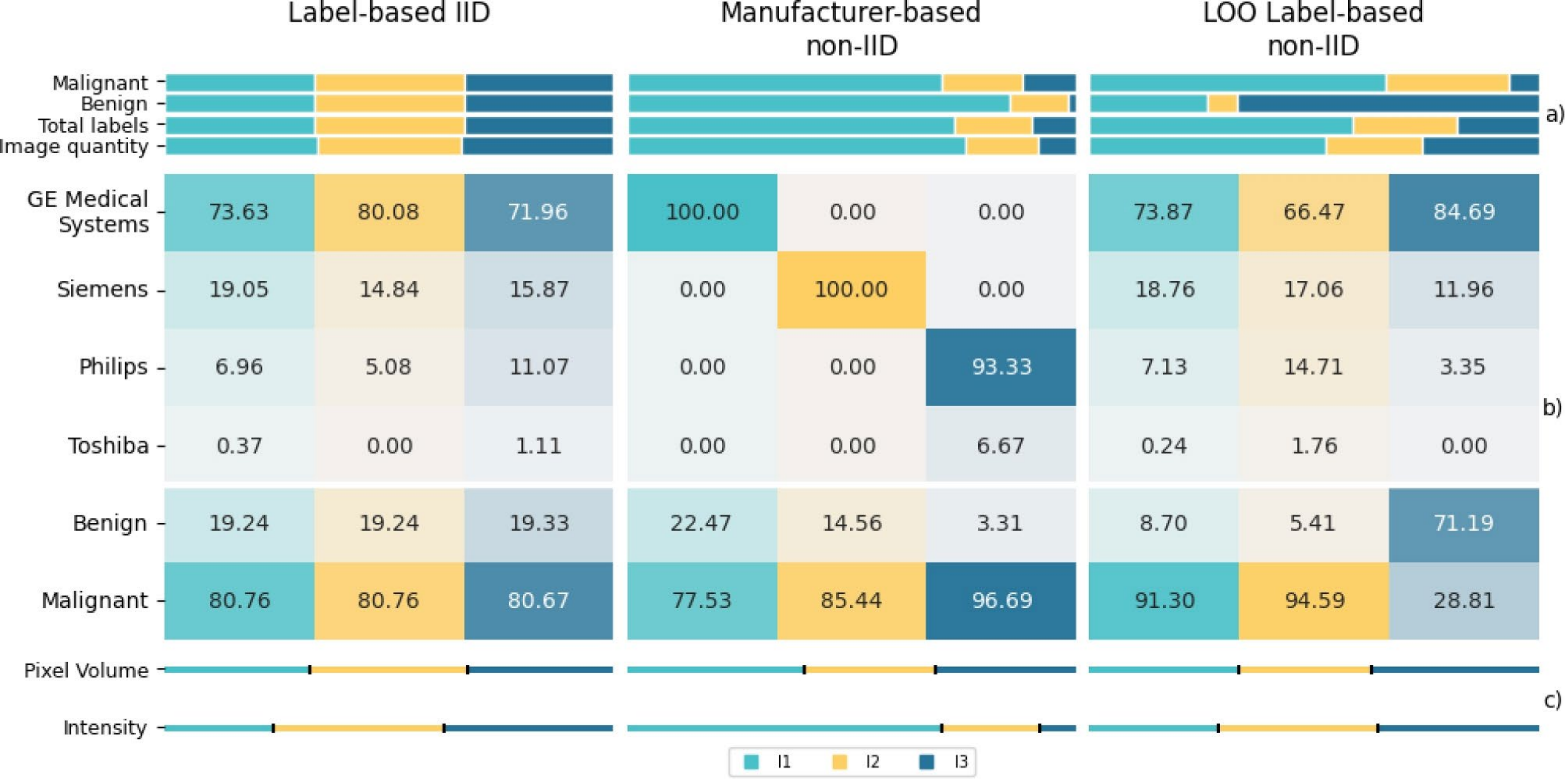
Data distribution characteristics in the **LUng Nodule Analysis 2016 (Luna16)** dataset: distributed label-based IID, manufacturer-based non-IID and LOO (leave-one-out) label-based non-IID data for each individual institution’s training dataset (I1, I2, I3). (**a**) displays the amount of images, total, benign and malignant labels when distributing the original Luna16 training dataset. (**b**) shows the manufacturer and label compositions. (**c**) indicates the relative sizes of the median box volumes and intensity values compared to sum of the corresponding medians. The smaller the part of a bar associated with a particular institution compared to the other parts, the smaller the corresponding median.

The allocation of images and labels for each institution’s training dataset in these distributions and the individual dataset compositions are illustrated in Fig. 2. A qualitative sample of selected images highlighting the differences between different manufacturers can be found in Supplementary Fig. S1 While the label-based IID distribution allocates equal amounts of images and labels for each dataset, the manufacturer-based non-IID and LOO label-based non-IID distributions result in skewed images and label assignments.

In the label-based IID and LOO label-based non-IID distributions, we observe nearly homogeneous manufacturer compositions among datasets. The manufacturer-based non-IID distribution, intentionally designed to create datasets with disjoint manufacturer sets, introduces a slight label skew. Conversely, the label-based IID distribution exhibits a homogeneous label composition across datasets, while the LOO label-based non-IID distribution results in a notable difference in label composition among datasets from Institution 1 (I1), Institution 2 (I2) compared to those from Institution 3 (I3).

No substantial differences among the datasets of the different data distributions were observed for mean spacings and the box volume distribution. Only the manufacturer-based non-IID data distribution shows larger differences between the median intensity values of the different institutions.

#### Model performance

**Fig. 3:**
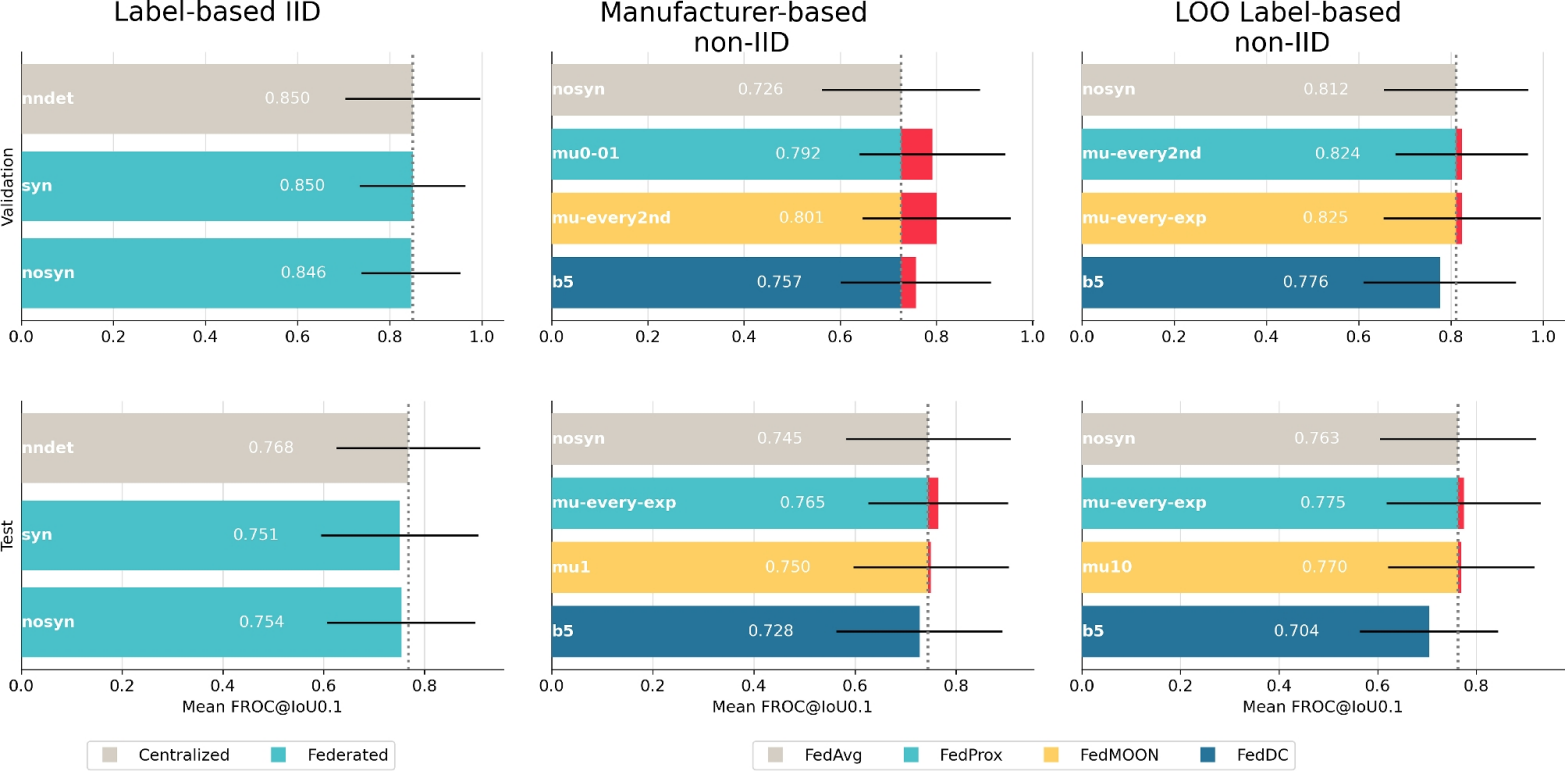
Performance comparison in the **LUng Nodule Analysis 2016 (Luna16)** dataset: Mean FROC at an IoU threshold of 0.1 based on bootstrapping with 1000 repetitions for different data distributions. Validation and test performance shown in top and bottom row respectively. Label-based IID data distribution compares centalized nnDetection to the two FedAvg version while the non-IID experiments compare FedAvg nosyn to the best-performing alternative FL strategies. The black bars indicate the 95% confidence intervals.

The results of the model performance evaluation are shown in Fig. 3. Label-based IID data distribution experiments compare centralized nnDetection against FedAvg utilizing the two distinct types of data fingerprints *syn* and *nosyn*. Results on the validation set show that federated models perform on par with centralized nnDetection. On the test set, all models experience performance drops, with federated models exhibiting slightly worse performance than centralized nnDetection.

The non-IID data distributions compare the *nosyn* FedAvg version to alternative FL strategies such as FedProx, FedMOON, and FedDC. *Syn* and *nosyn* refer to the type of data fingerprint used during the self-configuration process preceding the model training. The choice of the *nosyn* version aims to facilitate a comparison with centralized nnDetection. A more detailed explanation regarding *syn* and *nosyn* FedAvg and the data fingerprint can be found in Section Global Data Fingerprint.

In both the manufacturer-based and the LOO label-based non-IID data distributions, validation performance declines compared to the label-based IID data distribution. From the two non-IID approaches, the manufacturer-based distribution resulted in a larger performance drop compared to the label-based counterpart.

While the alternative FL strategies show substantial validation performance improvements in the manufacturer-based non-IID data distribution compared to FedAvg, the improvements observed in the LOO label-based non-IID distribution are minor in comparison.

On the test set, only FedProx and FedMOON achieve a slight performance improvements compared to FedAvg for both non-IID data distributions. Except for FedAvg in the manufacturer-based non-IID data distribution, all models exhibit a performance drop on the non-IID test sets. However, the test performance of FedAvg, FedProx and FedMOON in the non-IID data distributions is similar or better than the test performance of FedAvg in the IID data distribution.

### Duke breast cancer MRI dataset

The Duke dataset comprises images from two distinct device manufacturers, thereby limiting its manufacturer-based data distribution to two datasets. Due to the presence of malignant labels only, a non-IID distribution based on labels is not possible. Instead, experiments for a label-based IID and manufacturer-based non-IID data distribution are conducted. In contrast to the Luna16 data, the training and test datasets vary across different data distributions. This approach is adopted to maximize the sizes of the training datasets, given the limited number of virtual institutions.

#### Data distribution comparison

For the label-based IID distribution, the training dataset is evenly split, with each institution receiving 50% of the images. The manufacturer-based non-IID distribution, allocates images based on the proportions associated with manufacturers GE Medical Systems and Siemens to the institutions.

**Fig. 4:**
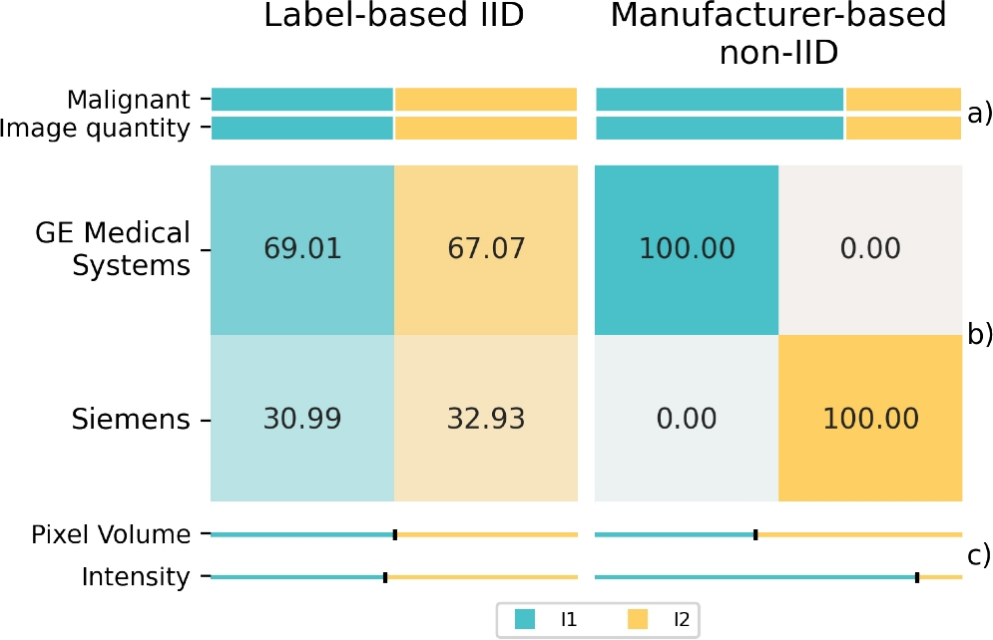
Data distribution characteristics in the **Duke Breast Cancer MRI (Duke)** dataset: distributed label-based IID and manufacturer-based non-IID for the training datasets of the two institutions (I1, I2). (**a**) displays the amount of images and malignant labels when distributing the original Duke training dataset. (**b**) shows the manufacturer composition. (**c**) indicates the relative sizes of the median box volumes and intensity values compared to sum of the corresponding medians. The smaller the part of a bar associated with a particular institution compared to the other parts, the smaller the corresponding median.

The resulting quantity skews and manufacturer compositions are illustrated in Fig. 4. The stacked bar plots show the allocation of images and labels to each institution for the two data distribution strategies. Below these plots, the heat maps display the manufacturer composition of the individually distributed datasets. The label-based IID data distribution results in two datasets with similar manufacturer compositions, whereas the manufacturer-based data distribution leads to a skew in the quantity of images and labels between the two datasets.

Further analysis reveals minimal disparities in mean spacings or box volumes across the distributions and institutions. However, there are notable differences in the median intensities between the datasets among the two data distributions. Minor differences are observed in the label-based IID distribution, while substantial differences are evident in manufacturer-based non-IID distribution.

#### Model performance

**Fig. 5:**
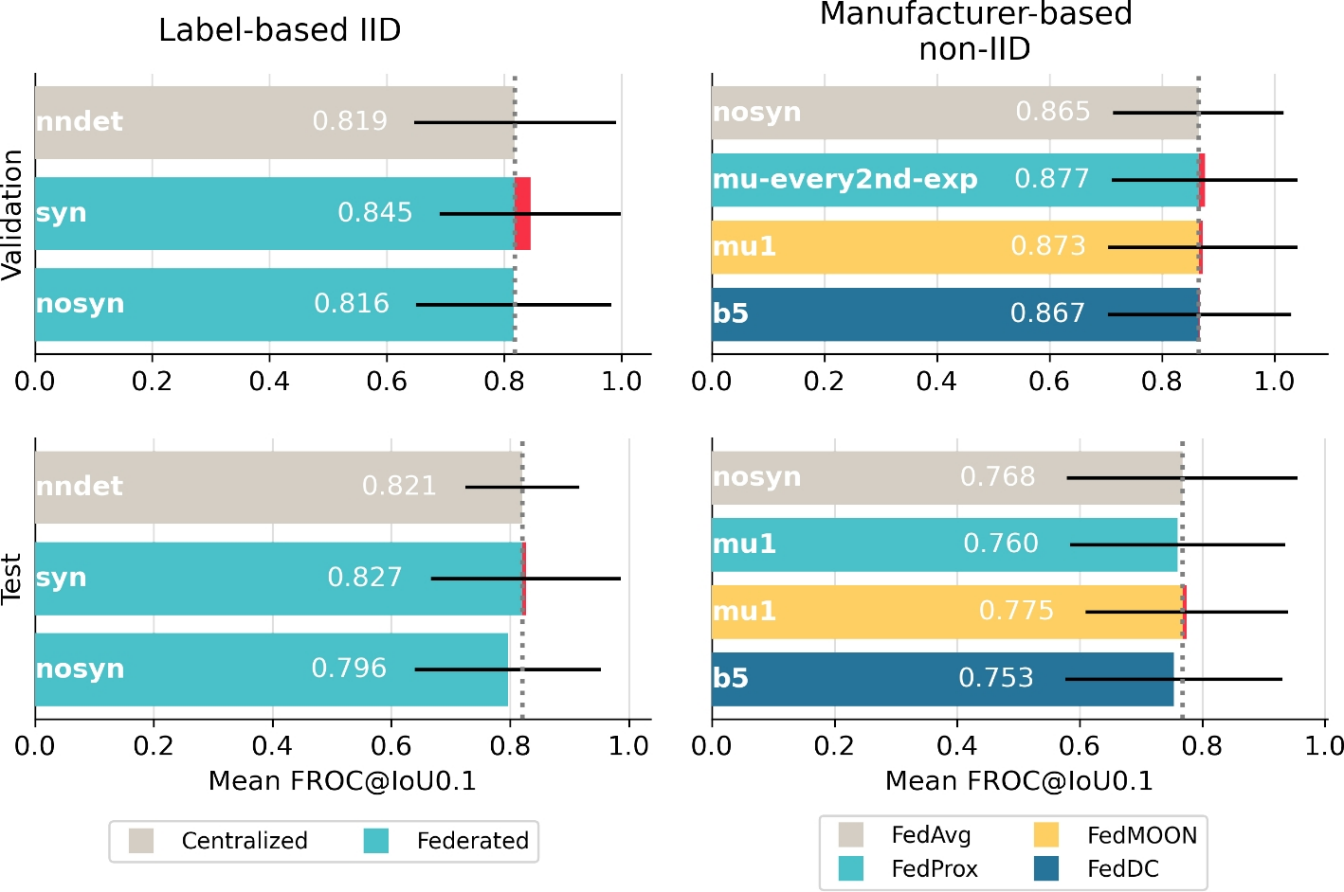
Performance comparison in the **Duke Breast Cancer MRI (Duke)** dataset: Mean FROC at an IoU threshold of 0.1 based on bootstrapping with 1000 repetitions for the two data distributions. Validation and test performance are shown in top and bottom row respectively. Label-based IID data distribution compares centalized nnDetection to the two FedAvg version while the manufacturer-based non-IID experiments compare FedAvg nosyn to alternative FL strategies. The black bars indicate the 95% confidence intervals.

Label-based IID and manufacturer-based non-IID model performance results are presented in Fig. 5. A qualitative sample selection is included in the supplementary information in Supplementary Fig. S2. The validation performances on Duke’s label-based IID data show that FedAvg performs comparably to centralized nnDetection. However, FedAvg shows a minor peformance drop on the test set, resulting in a slight decrease in performance compared to centralized nnDetection, particularly for the nosyn FedAvg configuration. Additionally, the confidence interval of centralized nnDetection test is notablely smaller compared to the confidence interval of FedAvg.

The performance on the validation set of the manufacturer-based non-IID data distribution shows an unexpected performance improvement for the nosyn FedAvg strategy compared to the IID scenario. Consequently, even the best-performing alternative FL strategy achieves only marginal improvements or performs worse compared to FedAvg. In contrary to the IID scenario, the models in the non-IID scenario experience a substantial performance drop on the test set compared to the validation set.

## Discussion

Our study demonstrates the feasibility of a federated self-configuring MOD framework aimed at addressing data scarcity arising from strict data privacy regulations, while also identifying challenges encountered and opportunities for improvement.

In the Luna16 dataset, various aggregation strategies (FedAvg, FedProx, FedMOON) performed similarly or better in the non-IID setting compared to the conventional aggregation algorithm FedAvg in IID, while not being far behind centralized non-federated training. This finding suggests a promising generalization of the models trained on the non-IID datasets.

The experiments with the Duke dataset involved only two clients, resulting in model performance that was approximately 5-7% lower in the non-IID scenario compared to the IID scenario. Further investigation revealed that the manufacturer-based non-IID data distribution significantly improved validation performance for Institution 2 compared to the IID scenario. However, this improvement can be attributed to the fact that Institution 2 has a much smaller dataset than Institution 1, leading to overfitting in its local model and consequently lower test performance.

Studying non-IID distributions in the Duke dataset was essential, as the MR data they contain are less prone to standardization compared to CT data, highlighting clearer differences between manufacturers. This suggests that larger datasets with more clients could potentially yield better results in non-IID scenarios. Although more aggressive data augmentation techniques could improve performance and reduce overfitting, they were beyond the scope of this study. Our primary focus was to demonstrate the feasibility of various federated learning strategies using a self-configuring method, such as nnDetection, in an out-of-the-box manner.

Experiments with the Luna and Duke datasets show relatively wide confidence intervals, which is also observed in the centralized models. Hence, direct conclusions regarding which aggregation method performs best for each scenario could not be made. This study is meant to serve as a basis for the aggregation and fingerprinting methods.

Based on our experiments with non-IID data, it becomes clear that monitoring the characteristics of the individual clients can help identify potential local overfitting issues. Identifying manufacturer-based non-IID distributions can prompt to enhancing data augmentation, while label-based non-IID could hint to balancing strategies. Further, federated averaging, while typically being the baseline aggregation strategy, performed comparably to the alternatives, and we would recommend it as a starting point even in complex scenarios. We further recommend using synthetic properties with samples drawn from Gaussian distributions for data fingerprinting, as we found it to perform comparably well to more data heavy approaches, while posing no data leakage risk.

One of the key challenges in implementing a distributed self-configuration process for a federated extension of nnDetection was minimizing the exchange of patient data during the creation of global data fingerprint. In particular, the intensity distribution feature of the data fingerprint relies on voxel samples from images. Recomputing intensity-related attributes on the server when creating the global data fingerprint requires sending voxel samples from local datasets to the server. This introduces a vulnerability that could potentially be exploited by attackers, and suggests the investigation of alternative approaches. Possible opportunities for improvement comprise reducing the exchange of patient-related data during the distributed self-configuration process by creating a less accurate data fingerprint. In this context, implementing additional security features, including the Transport Layer Security (TLS) protocol, enables experiments in real-world scenarios. Sending the sampled voxels in a single transfer after random shuffling their order can also serve as a practical solution. Additionally, the sweep post-processing function of nnDetection, responsible for optimizing inference parameters, was not trivial to refactor, as it required that the clients send their predictions to the central server to compute the final global evaluation scores.

There are various methods for creating non-IID datasets that simulate real-world usage, extending beyond labels and manufacturers to include factors like image and population characteristics, which can create more realistic testing environments. Additionally, selecting an open-source federated learning framework was challenging due to the diverse features offered by different options. Ultimately, Flower was chosen for its lightweight nature, intuitive usability, comprehensive documentation, and support for SSL/TLS-enabled connections.

The integration of FL into nnDetection is inspired by the work of Kades et al., who implemented an FL version of nnU-Net into the collaborative research platform Kaapana^25^. Since nnU-Net is the counterpart of nnDetection for segmentation tasks, they also had to address the challenge of a distributed self-configuration process. Although their experiments included only one dataset utilizing FedAvg as the FL strategy, their federated models yielded results on par with the centralized nnU-Net, motivating the development of a self-configuring FL MOD model. Since the experiments conducted by Parekh et al.^8^ investigating the potential of MOD in multi-domain, multi-task settings produced encouraging results, our study aims to provide a more comprehensive investigation of FL for multi-task MOD. Therefore, we use nnDetection, which provides a multi-task MOD platform incorporating MOD architectures such as Retina U-Net. Additionally we implement and evaluate different FL strategies, assessing the impact of data domain shifts on the performance of FL models. The results of our experiments are in line with Parekh et al.’s findings and confirm the potential of FL in MOD.

Overall, this work serves as a proof-of-concept for MOD and facilitates further research in this field. It implements a fully automatic federated self-configuring approach that could enable fast prototyping and usage, even by non-technical users. We distribute our work in an open-source format and make a Docker image available, fostering the integration into existing federated collaboration.

Future work could include larger multi-site cohorts that can better mirror real-world usage and cover differences in populations and equipment more appropriately. Such experiments can provide a better understanding of the impact of data domain shifts on FL model performance. Consequently, these experiments can promote the development of FL strategies specifically tailored to the unique requirements of MOD. Lastly, different FL frameworks can be tested to compare their results as well as their effectiveness in large deployments. Integration of this work into FL systems like Kaapana could further enhance its applicability and foster research to create more robust MOD algorithms.

This project presents an implementation of self-configuring MOD with FL. It combines the advantages of training state-of-the-art MOD models without manual intervention, while also granting access to a large amount of data through FL. The conducted experiments yield promising results and encourage further investigation in real-world settings.

## Methods

### Datasets

The LUng Nodule Analysis 2016 dataset (Luna16) is a subset of the Lung Image Database Consortium (LIDC) dataset^26^, originally curated for medical image segmentation. Ethics approval for this public dataset was provided by each participating institution’s IRB. All participants gave informed consent. All research involving human participants adhered to the declaration of Helsinki, relevant guidelines, and regulations. It comprises pulmonary nodules with a minimum radius of 3mm (box volume median: 847 voxels), which are identified and verified by at least three experts. Non-nodular regions, nodules less than 3mm in radius, and nodules detected by one or two radiologists are excluded from the dataset as unrelated findings. Training data covers the initial eight subsets of Luna16 data, while the last subset is reserved for testing. One case is excluded due to an abnormal Hounsfield unit range. More than three-quarters of the cases are malignant, predominantly originating from the manufacturer GE Medical Systems. The mean spacings for the x and y dimensions are 0.69mm, while the z dimension is 1.57mm. The intensity value distribution ranges from -1024 to 2468 Hounsfield Units (HU), with a median intensity value of -101 while values below and above the 0.5th and 99.5th percentiles are considered as outliers.

The Duke Breast Cancer MRI dataset (Duke)^17,18^ comprises 922 high-resolution MRIs, including comprehensive metadata annotated by medical experts. Ethics approval for this public dataset was provided by the Duke University Health System IRB. All participants gave informed consent. All research involving human participants adhered to the declaration of Helsinki, relevant guidelines, and regulations. Key characteristics of the dataset include mean spacings of 1.4 for the x and y dimensions, and 2.0 for the z dimension. Intensity values span from -4671 to 10834 (0,5th - 99.5th percentile), with a median value of 96. Compared to Luna16, the Duke dataset’s images exhibit larger mean spacing and larger boxes, with a median size of 5460 voxels, all of which are malignant and originate from devices of only two manufacturers.

### nnDetection

nnDetection^12^ is a state-of-the-art self-configuring MOD method that adjusts itself to novel datasets, with successful applications^27,28^. It automatically selects optimal training parameters based on a set of rule-based parameters depending on a data fingerprint and fixed parameters. The data fingerprint characterizes the training data through predefined properties, such as intensity value distribution or object sizes. The selection process for rule-based parameters leverages these data characteristics, optimizing parameters like anchor sizes based on object sizes in the training dataset. Fixed parameters remain unchanged unless manually adjusted, relying on heuristics and empirical evaluations. Following the training procedure, nnDetection further refines object detection hyperparameters, such as the minimum object size, through an automatic empirical optimization. Starting from set of predefined values the empirical hyperparameter optimization refines those initial values sequentially due their interdependencies.

#### Global data fingerprint

The creation of a global data fingerprint is an important aspect of the federated self-configuring MOD framework developed in this project. The data fingerprint characterizes the dataset with a set of specific properties. In a federated setting, multiple local data fingerprints are generated based on the local dataset of each client. Those local data fingerprints need to be aggregated into a single global data fingerprint to generate a plan that facilitates the generation of a common global model.

Most properties of the data fingerprint are based on individual images of the local datasets and can be easily concatenated in the global data fingerprint. However, properties such as the intensity value distribution, which are based on all images in local datasets, pose a significant challenge. The intensity value distribution from each local dataset cannot be concatenated in the global data fingerprint because the intensity value distribution of the distributed global dataset needs to be represented by a single global property. Since these properties from the local datasets include values such as percentiles and standard deviations, there is no straightforward algorithm to reduce multiple of those values into a single value. To address this issue, we evaluate two options:

- **nosyn** involves sampling every 10th voxel of each image from the datasets of all clients, sending them to the server, and recomputing corresponding intensity value distribution properties. While this option offers good accuracy similarly to centralized nnDetection, it exposes patient data to the network and the server.
- **syn** proposes computing these properties using samples drawn from Gaussian distributions, using intensity properties from individual images of local datasets. For each image in the distributed global dataset, a truncated Gaussian distribution with the mean and standard deviation of the corresponding intensity value distribution within the range of the minimum and maximum values is created. The global intensity property in the global data fingerprint is then recomputed based on samples drawn from each of those Gaussian distributions. This approach prioritizes data privacy and empirical tests have shown no performance loss with this option in the label-based IID scenarios (Fig. 3, Fig. 5).

However, to enable a better comparison with centralized nnDetection, non-IID experiments utilize nosyn data fingerprints since they are more similar to centralized nnDetection’s data fingerprint.

#### Empirical hyperparameter optimization

The empirical hyperparameter optimization (sweep) refines parameters as the minimum object size or the the minimum prediction score threshold. Starting from a set of predefined default values each of the sweep parameters is sequentially optimized as interdependencies prevent a parallel optimization.

For each predefined value of a particular sweep parameter, the corresponding predictions for each image are evaluated based on a defined target metric such as the FROC score. The predefined value producing the best results is subsequently selected and updated in the hyperparameter configuration before the next sweep parameter is optimized.

Due to the interdependencies among the sweep parameters and the FROC score serving as the target metric, conducting an independent local empirical optimization of the clients in a federated setup with subsequent aggregation of results on the server side is not feasible. Instead, for each sweep parameter along with its default value set, the clients conduct an empirical optimization and send prediction matches (predictions matching ground-truth boxes) and prediction scores to the server. This step is necessary as FROC scores from different clients rely on different thresholds and cannot be aggregated.

With the received prediction matches and prediction scores, the server computes the final FROC scores for each value of the corresponding sweep parameter and updates the hyperparameter configuration accordingly before initiating the optimization of the next sweep parameter. Given the high cost associated with this empirical hyperparameter optimization, it is performed only once after completing the final global training round.

### Federated Learning

FL allows joint training of a global model that benefits from a large distributed training data pool across different clients (institutions). Each client trains a local model based on their local dataset and submits only the resulting model weights to the server to preserve patient data privacy.

The standard FL approach to aggregate model weights from different clients is Federated Averaging (FedAvg)^2^, being especially effective when dealing with homogeneously distributed data among clients (IID). As FedAvg employs weighted averaging, it is also well-suited to handle quantity skews^19^. However, FedAvg faces challenges when faced with data domain shifts, where data distributions among clients are heterogeneous (non-IID). This heterogeneity arises commonly in real-world settings, where guidelines, imaging devices, and protocols vary among different institutions. As a result, loss divergence and performance degradation become issues.

To alleviate these challenges, alternative FL strategies have been developed, like FedProx^4^, FedMOON^13^, and FedDC^14^. FedProx and FedMOON address the problem by introducing additional proximal terms to local loss functions. These terms encourage the local models to remain closer to the most recent global model, with the extent of this proximity determined by the hyperparameter $\mu$. Equations 1 and 2 delineate the specifics of the respective adjustments to local loss functions. FedProx’s proximal term calculates the residual between the most recent global model *w* and the current local model. The greater the divergence of the local model $w^r$ from the global model, the larger the penalty imposed by the proximal term. The experiments of Quinbin Li et al.^19^ show that FedProx achieves particularly good results in label distribution skews such as the label-based non-IID distribution in this project. Similarly, FedMOON incorporates the more sophisticated, albeit computationally expensive, model-contrastive loss into the local function, where $\tau$ represents a temperature parameter. In contrast to only considering the global model $z_{glob}$, the model-contrastive loss also encompasses the local model from the previous epoch, denoted as $z_{prev}$. Using a similarity metric (e.g., cosine similarity), the objective is to increase the difference between the current local model *z* and $z_{prev}$ while decreasing the difference to $z_{glob}$.

1 $$\begin{aligned}&Loss(w(x), y) + \frac{\mu }{2} \cdot \underbrace{||w - w^{r}||^2}_{\text {proximal term}} \end{aligned}$$

2 $$\begin{aligned}&Loss(w(x), y) -\mu \cdot \underbrace{\log \frac{\exp (\text {sim}(z, z_{glob})/\tau )}{\exp (\text {sim}(z, z_{glob}/\tau )+\exp (\text {sim}(z,z_{prev})/\tau )}}_{\text {model-contrastive loss}} \end{aligned}$$

FedDC, on the other hand, has been specifically developed for a large number of clients with small datasets executing only a very few local epochs. It applies FedAvg within specific intervals of global rounds, and otherwise randomly swaps local model weights among different institutions without weight aggregation. Compared to FedProx and FedMOON, FedDC is much less computationally demanding, offering an opportunity to mitigate the negative impact of data domain shifts on model performance at low cost.

#### Flower

The implementation applies nnDetection in a simulated decentralized setup using the FL framework Flower. Alternative FL frameworks that have also been considered during the course of this project include NVFlare^29^, FedML^30^, and PySyft^31^.

Flower is an open-source FL framework designed for heterogeneous edge devices, compatible with PyTorch and TensorFlow. It supports customizable strategies and client classes, simplifying setup while allowing for tailored configurations.

The selection of Flower for this project is driven by its lightweight nature, ease of use, and ability to accommodate the aforementioned customization options. Moreover, it comes equipped with pre-implemented FL strategies, while also offering the infrastructure for straightforward implementation of other FL strategies.

### Model training

All models are trained for ten global rounds with five local epochs each. Training with the Luna16 dataset uses hard negative mining (HNM), as it showed better stability throughout training compared to the focal loss. Conversely, the training for Duke dataset relies on the focal loss, due to its superior performance in centralized training scenarios and good stability.

#### Hard negative mining

Hard negative mining (HNM) addresses imbalanced datasets in object detection by identifying and prioritizing challenging negative samples during training. When the number of negatives outweighs positives, models can become biased towards predicting negatives. HNM evaluates the model predictions on negatives, identifies misclassifications or high-confidence errors (hard negatives), and adjusts the training process to assign higher importance to these hard negatives, usually by re-weighting the loss function. This encourages the model to learn from errors and improve the discrimination between true and false positives. Although not attributed to a single publication, HNM is employed by popular object detection algorithms like^32^ and^33^.

#### Focal loss

The focal loss^34^, akin to HNM, addresses the challenge of imbalanced datasets by prioritizing hard-to-classify samples. It modifies the standard cross-entropy loss function, decreasing the impact from well-classified examples while increasing the impact from poorly classified ones.

3 $$\begin{aligned} \text {p}_\text {t}, \alpha = {\left\{ \begin{array}{ll} \text {p}, \alpha _\text {t} & y=1\\ 1 -\text {p}, 1 - \alpha & \text {otherwise} \end{array}\right. } \end{aligned}$$

4 $$\begin{aligned} \text {Focal Loss}&= -\alpha _\text {t}(1-\text {p}_\text {t})^\gamma \text {log}(\text {p}_\text {t}) \end{aligned}$$

The focal loss computation is given by Eq. (4). Based on the sample’s class, the hyperparameter $\alpha _t \in [0, 1]$ (Equation 3) weights the loss. The critical component of the focal loss is the term $(1-\text {p}_t)^\gamma$, which reduces the impact of well-classified positive or negative samples. When $y=1$, as $\text {p}_t$ (Eq. 3) approaches 1 and the larger the hyperparameter $\gamma$, the impact on the loss of the current prediction decreases. The same is true for $y=0$ and p approaching 0.

### Evaluation

#### Free-response receiver operating characteristic score

The Free-Response Receiver Operating Characteristic (FROC) score^35^ is a widely-employed metric for evaluating model performance in MOD. It compares the true positive rate (TPR) [0, 1] (Eq. 5) against the average false positives per image (FPPI) $[0, \infty ]$ (Eq. 6) across multiple prediction score thresholds at a fixed IoU threshold.

5 $$\begin{aligned} \text {TPR}&= \frac{\text {TP}}{\text {TP} + \text {FN}}\end{aligned}$$

6 $$\begin{aligned} \text {FPPI}&= \frac{\text {FP}}{\text {total images}} \end{aligned}$$

The FROC curve is often reduced to a single value by computing the average TPR at different FPPI values. In general, the FROC score is useful for focusing on the detection of true positive samples and finding the optimal ratio between the TPR and FPPI across various thresholds.

## Supplementary Information

Supplementary Figures.

## Data Availability

The publicly available LUng Nodule Analysis 2016 dataset^15^ and the Duke Breast Cancer MRI dataset^16–18^ were used for conducting the experiments of this project.
