# Supplementary material for: The potential of federated learning for self-configuring medical object detection in heterogeneous data distributions
Source: Sci Rep. 2024 Oct 11;14:23844. doi: 10.1038/s41598-024-74577-0 (PMC11470020; doi:10.1038/s41598-024-74577-0)
Supplement: Supplementary file 1 — Supplementary Figures. [file 41598_2024_74577_MOESM1_ESM.pdf]

The Potential of Federated Learning for Self-Configuring Medical Object Detection in Heterogeneous Data Distributions

Gabriel Rashidi[1,2], Dimitrios Bounias[1,3,*], Markus Bujotzek[1,3], Andrés Martínez Mora[1,3], Peter Neher[1,4,5], and Klaus H. Maier-Hein[1,2,3,4,5,6]

[1]German Cancer Research Center (DKFZ) Heidelberg, Division of Medical Image Computing, Heidelberg, 69120, Germany
[2]Faculty of Mathematics and Computer Science, Heidelberg University, Heidelberg, 69120, Germany
[3]Medical Faculty Heidelberg, Heidelberg University, Heidelberg, 69120, Germany
[4]German Cancer Consortium (DKTK), Partner Site Heidelberg, Im Neuenheimer Feld 280, 69120 Heidelberg, Germany
[5]Pattern Analysis and Learning Group, Heidelberg University Hospital, Heidelberg, 69120, Germany
[6]National Center for Tumor Diseases (NCT), Heidelberg University Hospital (UKHD) and German Cancer Research Center (DKFZ), Im Neuenheimer Feld 460, 69120 Heidelberg, Germany
[*]dimitrios.bounias@dkfz-heidelberg.de

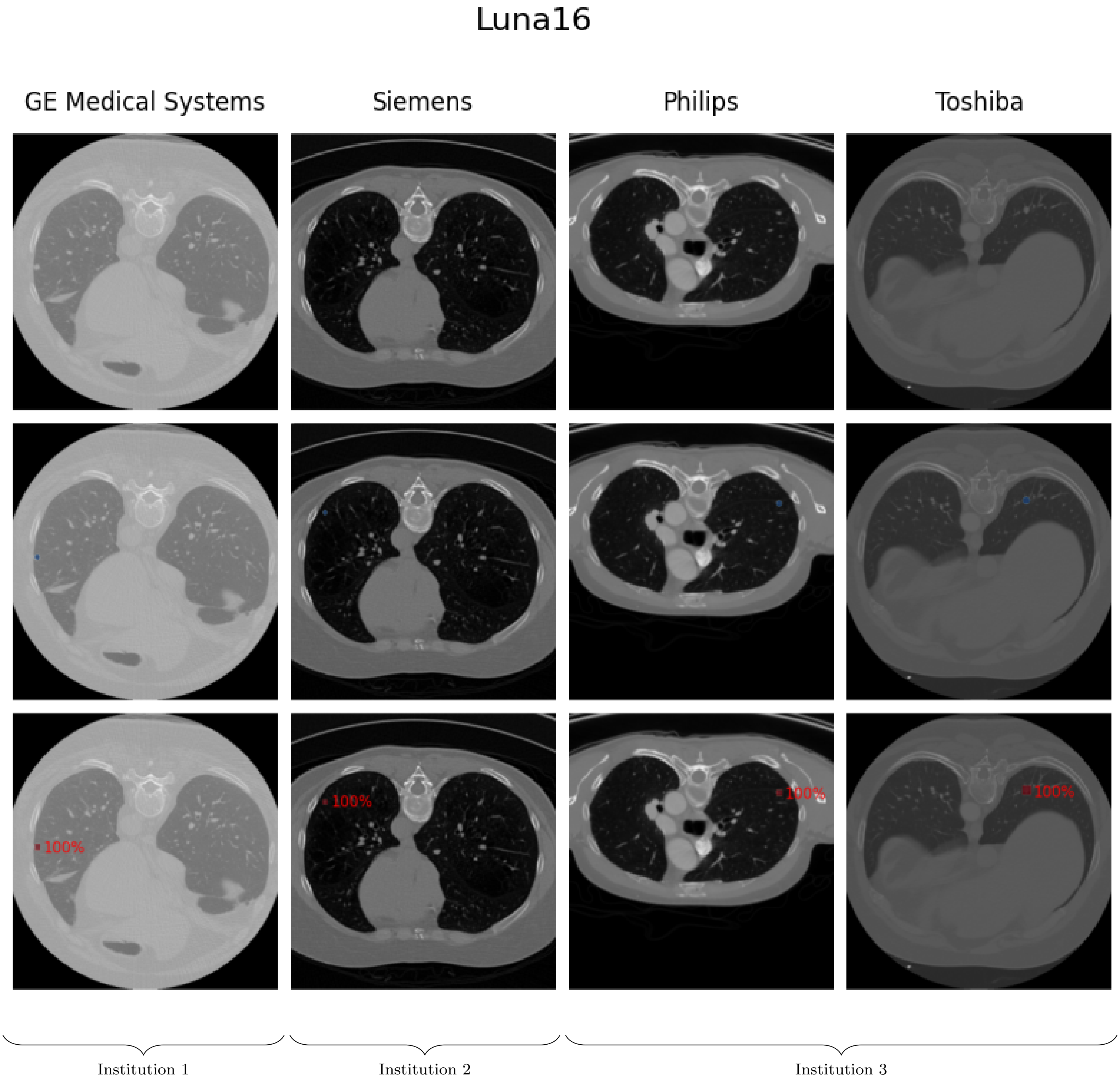

Figure 1: Qualitative sample cases of the different device manufacturers in the Luna16 dataset. The middle and lower row include the ground truth (blue) and predicted (red) bounding boxes respectively.

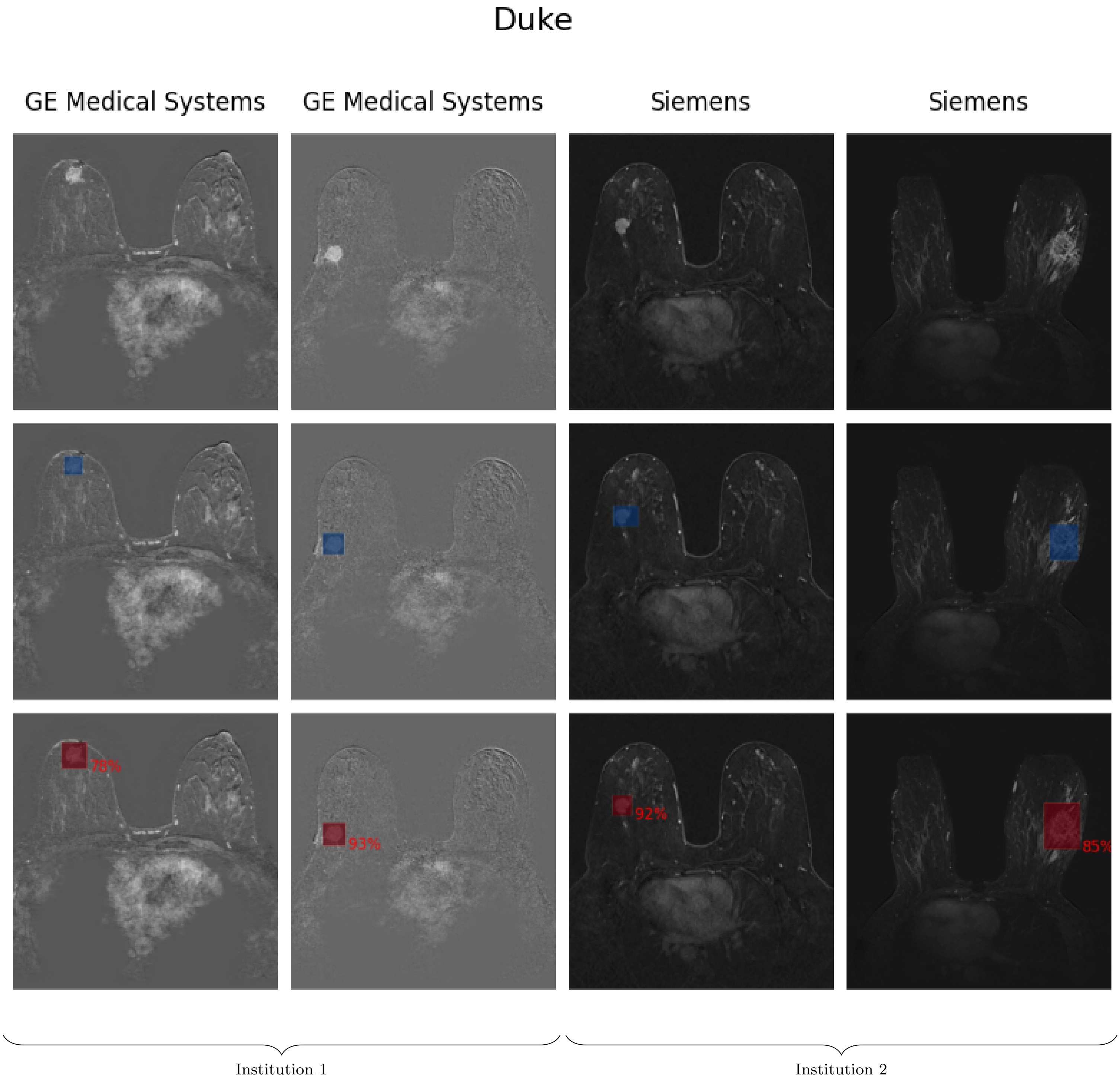

Figure 2: Qualitative sample cases of the different device manufacturers in the Duke dataset. The middle and lower row include the ground truth (blue) and predicted (red) bounding boxes respectively.
